# Reclassification of aortic stenosis by fusion of echocardiography and computed tomography in low-gradient aortic stenosis

**DOI:** 10.1007/s12471-020-01501-2

**Published:** 2020-10-14

**Authors:** N. El Faquir, M. E. Vollema, V. Delgado, B. Ren, E. Spitzer, M. Rasheed, Z. Rahhab, M. L. Geleijnse, R. P. J. Budde, P. P. de Jaegere, J. J. Bax, N. M. Van Mieghem

**Affiliations:** 1 Department of Cardiology, Thoraxcenter, Erasmus University Medical Centre, Rotterdam, The Netherlands; 2 Department of Cardiology, Leiden University Medical Centre, Leiden, The Netherlands; 3 Department of Radiology and Nuclear Medicine, Erasmus University Medical Centre, Rotterdam, The Netherlands

**Keywords:** Aortic stenosis, Transcatheter aortic valve replacement, Multimodal imaging

## Abstract

**Background:**

The integration of computed tomography (CT)-derived left ventricular outflow tract area into the echocardiography-derived continuity equation results in the reclassification of a significant proportion of patients with severe aortic stenosis (AS) into moderate AS based on aortic valve area indexed to body surface area determined by fusion imaging (fusion AVA_i_). The aim of this study was to evaluate AS severity by a fusion imaging technique in patients with low-gradient AS and to compare the clinical impact of reclassified moderate AS versus severe AS.

**Methods:**

We included 359 consecutive patients who underwent transcatheter aortic valve implantation for low-gradient, severe AS at two academic institutions and created a joint database. The primary endpoint was a composite of all-cause mortality and rehospitalisations for heart failure at 1 year.

**Results:**

Overall, 35% of the population (*n* = 126) were reclassified to moderate AS [median fusion AVAi 0.70 (interquartile range, IQR 0.65–0.80) cm^2^/m^2^] and severe AS was retained as the classification in 65% [median fusion AVAi 0.49 (IQR 0.43–0.54) cm^2^/m^2^]. Lower body mass index, higher logistic EuroSCORE and larger aortic dimensions characterised patients reclassified to moderate AS. Overall, 57% of patients had a left ventricular ejection fraction (LVEF) <50%. Clinical outcome was similar in patients with reclassified moderate or severe AS. Among patients reclassified to moderate AS, non-cardiac mortality was higher in those with LVEF <50% than in those with LVEF ≥50% (log-rank *p* = 0.029).

**Conclusions:**

The integration of CT and transthoracic echocardiography to obtain fusion AVAi led to the reclassification of one third of patients with low-gradient AS to moderate AS. Reclassification did not affect clinical outcome, although patients reclassified to moderate AS with a LVEF <50% had worse outcomes owing to excess non-cardiac mortality.

## What’s new?

- Fusion of Doppler echocardiography and computed tomography in patients who underwent transcatheter aortic valve implantation (TAVI) for low-gradient, severe aortic stenosis (AS) (<40 mm Hg) resulted in the reclassification of 35% of patients to moderate AS.
- The composite endpoint of all-cause mortality and heart failure rehospitalisations post-TAVI was similar in patients reclassified to moderate AS and patients with severe AS.
- Patients reclassified to moderate AS showed similar improvements in New York Heart Association class to those with severe AS.
- Non-cardiac death was more frequent in patients reclassified to moderate AS with LVEF <50% versus LVEF ≥50%.

## Introduction

Accurate diagnosis of the severity of aortic valve stenosis (AS) is pivotal to the decision as to whether to proceed with valve replacement therapy and has important prognostic implications [1, 2]. Isolated aortic valve replacement (AVR) or transcatheter aortic valve implantation (TAVI) is indicated in symptomatic severe AS but not in moderate AS according to current guidelines [1, 2]. Transthoracic echocardiography (TTE) is the imaging modality of choice to characterise and quantify aortic valve disease [1]. TTE provides a visual assessment of the aortic valve anatomy and relies on Doppler techniques to determine transvalvular velocities and calculate the aortic valve area (AVA) [1]. The continuity equation to calculate the AVA uses the premise that the left ventricular outflow tract (LVOT) is circular. However, the LVOT and aortic annulus resemble more an ellipse [3, 4]. In addition, the operator dependency of TTE analysis and suboptimal acoustic windows may result in important measurement inaccuracies that may mislead clinical judgement and treatment decisions. Multi-slice computed tomography (MSCT) is a three-dimensional imaging tool that offers an accurate appreciation of the elliptic morphology and dimensions of the LVOT and aortic annulus [5].

Recently proposed fusion imaging techniques combine the LVOT dimensions determined by MSCT with Doppler measurements obtained via TTE in the continuity equation formula, which theoretically leads to more accurate AVA measurement. Fusion imaging may help to assess the AS severity in cases where there are discrepancies in TTE findings, especially in the context of low AVA in combination with a low gradient (mean gradient <40 mm Hg) [6]. The aim of this study was to evaluate AS severity by a fusion imaging technique in patients with low-gradient AS and to compare the clinical impact of reclassified moderate AS versus severe AS after TAVI.

## Methods

### Patient population

We included consecutive patients who underwent TAVI for low-gradient (mean gradient <40 mm Hg), severe AS as assessed by TTE at the Leiden University Medical Centre (LUMC) and the Thoraxcenter, Erasmus University Medical Centre, The Netherlands (EMC), between April 2006 and September 2016. Patients without TTE and/or MSCT at baseline were excluded. A joint database was constructed including baseline demographics, procedural and clinical outcome data and selected imaging variables derived from TTE and MSCT. Incomplete clinical data were addressed by consulting referring physicians and patients whenever possible. Survival status was obtained from the Dutch Civil Registry. Written informed consent for the TAVI procedure and subsequent data analysis for research purposes was provided by every patient at the EMC. Both institutional review boards waived the need for patient written informed consent for retrospective analysis of clinically acquired data (EMC MEC no. 2019-0301). The study was conducted in accordance with the principles of the Declaration of Helsinki and did not fall under the scope of the Medical Research Involving Human Subjects Act according to the EMC Institutional Review Board.

### Transthoracic echocardiography

All patients underwent TTE (at baseline and 1 year after TAVI) in accordance with a standard protocol. Two-dimensional TTE and Doppler data were acquired with commercially available systems, Philips iE33 (Philips Medical System, Best, The Netherlands) or Vivid‑7 and E9 ultrasound systems (General Electric, Horten, Norway). Images were stored offline and all analyses were performed in accordance with current guidelines using the Image Arena workstation (TomTec Imaging System, Unterschleissheim, Germany) or EchoPac (112.0.1, GE Medical Systems, Horten, Norway) [7]. Mean aortic pressure gradient was obtained by tracing the continuous wave envelope [8]. The AVA was estimated by the continuity equation and divided by body surface area (BSA) to obtain the indexed AVA (AVAi) [1, 8]. The LVOT was defined 5 mm below the aortic annulus (parasternal long-axis view) and its area was calculated based on the measured LVOT diameter, assuming circularity. The left ventricular ejection fraction (LVEF) was either visually assessed or calculated with the modified Simpson method [9].

### Multi-slice computed tomography

Pre-procedural MSCT was performed in all patients with a dual source (Definition, FLASH or Force, Siemens Healthcare, Forchheim, Germany) or 64- and 320-detector row computed tomography scanner (Aquilion 64; Toshiba Medical Systems, Otawara, Japan and Aquilion ONE; Toshiba Medical Systems, Tochigi-ken, Japan) with electrocardiographic triggering or gated acquisitions in systole. CT scan settings for image acquisition were reported previously [6, 10]. All the reconstructions were stored on dedicated workstations for offline analysis (Vitrea 2, Vital Images, Plymouth, MN, USA and Intellispace, Philips, Best, The Netherlands). Aortic annulus and LVOT dimensions were analysed with 3Mensio software (Bilthoven, The Netherlands) and calcification was expressed using the score proposed by Rosenhek et al. and the Agatston calcium score [11, 12]. The LVOT was defined at the smallest area between 2 and 6 mm below the annular plane and measured by planimetry [13].

### Fusion imaging

Fusion implied the use of MSCT and TTE data in the continuity equation in order to reclassify AS severity. LVOT area measured by MSCT was used to replace LVOT area measured by TTE as described previously [6]. To calculate fusion AVAi the following formula was used [6]:

$$\text{Fusion AVAi}=(\frac{\boldsymbol{MSCT} \boldsymbol{LVOT} \boldsymbol{area} \boldsymbol{x} \boldsymbol{Echo} \boldsymbol{VTI} \boldsymbol{PW} \boldsymbol{LVOT}}{\boldsymbol{Echo} \boldsymbol{VTI} \boldsymbol{CW}\,\boldsymbol{Aortic}\,\boldsymbol{valve}})/\boldsymbol{body}\,\boldsymbol{surface}\,\boldsymbol{area}$$

where *VTI* = velocity time integral, *PW* = pulse wave Doppler and *CW* = continuous wave Doppler. Reclassification was based on fusion AVAi ≥0.6 cm^2^/m^2^ (moderate AS) and AVAi <0.6 cm^2^/m^2^ (severe AS).

### Statistical analysis

Normal distribution of continuous data was assessed by the Kolmogorov-Smirnov test. Values are expressed as mean ± SD or median (interquartile range, IQR) depending on distribution. Categorical data were presented as numbers and frequencies. Comparison of baseline characteristics was done by means of the Student *t*-test, Mann Whitney U test or chi-squared test. LVEF at baseline was compared with 1‑year follow-up LVEF after TAVI using the paired *t*-test. The primary endpoint was a composite of all-cause mortality and rehospitalisations for heart failure (HF) at 1 year. Cardiac mortality, non-cardiac mortality and rehospitalisations due to HF were secondary endpoints. Kaplan-Meier curves were used to assess the primary and secondary endpoints (30 days and 1 year) after TAVI. To further evaluate the impact of systolic LV function a separate analysis looked at differences in clinical endpoints between reclassified moderate and severe AS in patients with LVEF <50% and ≥50%.

Statistical significance was assumed when the *p*-value was <0.05. Statistical analysis was done using SPSS 24.0 (IBM Corporation, New York, NY, USA).

## Results

### Baseline characteristics

#### Overall cohort

The overall cohort consisted of 359 patients with low-gradient, severe AS on TTE; 57% were men with a median body mass index (BMI) of 26 (24–29) kg/m^2^ and a median logistic EuroSCORE of 16 (10–24) (Tab. 1). LVEF <50% at baseline was present in 57% of patients (*n* = 204). Median AVA was 0.8 cm^2^ (IQR 0.7–0.9 cm^2^), median AVAi was 0.4 cm^2^/m^2^ (IQR 0.4–0.5 cm^2^/m^2^), median mean gradient was 29 mm Hg (IQR 24–35 mm Hg) and median peak velocity was 3.5 m/s (IQR 3.2–3.9 m/s).

**Table 1:** Baseline characteristics of the overall cohort

	Overall cohort
	*n* = 359
*Demographics*	
Age (years), median (IQR)	80 (75–84)
Male, *n* (%)	206 (57)
Height (cm), median (IQR)	168 (162–175)
Weight (kg), mean ± SD	76 ± 14
Body mass index (kg/m^2^), median (IQR)	26 (24–29)
Body surface area (m^2^), median (IQR)	1.9 (1.7–2.0)
*Cardiac risk factors*	
Diabetes mellitus, *n* (%)	125 (35)
Hypertension, *n* (%)	271 (76)
*Medical history*	
Previous cerebrovascular accident or transient ischaemic attack, *n* (%)	75 (21)
Previous myocardial infarction, *n* (%)	107 (30)
Previous coronary artery bypass graft surgery, *n* (%)	110 (31)
Previous percutaneous coronary intervention, *n* (%)	157 (44)
Permanent pacemaker, *n* (%)	49 (14)
Peripheral vascular disease, *n* (%)	187 (52)
Chronic obstructive pulmonary disease, *n* (%)	104 (29)
Pulmonary hypertension, *n* (%)	60 (17)
Atrial fibrillation, *n* (%)	121 (34)
New York Heart Association class ≥III, *n* (%)	269 (75)
*Echocardiography*	
Left ventricular ejection fraction (%), mean ± SD	46 ± 15
Peak velocity (m/s), median (IQR)	3.5 (3.2–3.9)
Peak gradient (mm Hg), median (IQR)	49 (41–61)
Mean gradient (mm Hg), median (IQR)	29 (24–35)
Aortic valve area (cm^2^), median (IQR)	0.8 (0.7–0.9)
Indexed aortic valve area (cm^2^/m^2^), median (IQR)	0.4 (0.4–0.5)
LVOT VTI, median (IQR)	18 (15–21)
Aortic valve VTI, mean ± SD	79 ± 15
Stroke volume index, median (IQR)	32 (26–40)
Mitral regurgitation ≥ moderate, *n* (%)	84 (23)
Aortic regurgitation ≥ moderate, *n* (%)	51 (14)
*Multi-slice computed tomography*	
Annulus	
– Minimum diameter (mm), median (IQR)	22 (21–24)
– Maximum diameter (mm), median (IQR)	27 (26–29)
– Mean diameter (mm), median (IQR)	25 (23–26)
– Area (mm^2^), median (IQR)	464 (413–525)
– Perimeter (mm), median (IQR)	78 (74–84)
– Aortic root calcification ≥ moderate, *n* (%)	259 (72)
Left ventricular outflow tract	
– Area (mm^2^), median (IQR)	448 (389–515)
– LVOT calcification ≥ moderate, *n* (%)	46 (13)
Risk score	
– Logistic EuroSCORE, median (IQR)	16 (10–24)

Values are expressed as median (interquartile range, IQR), *n* (%) or mean ± SD

*LVEF* left ventricular ejection fraction, *LVOT* left ventricular outflow tract, *VTI* velocity time integral

#### Fusion reclassification

When integrating the MSCT-derived LVOT area into the continuity equation, the median AVA was 1.02 cm^2^ (IQR 0.87–1.21 cm^2^) and median AVAi 0.54 cm^2^/m^2^ (IQR 0.47–0.65 cm^2^/m^2^) (Tab. 2). Severe AS was retained as the classification in 65% of patients (*n* = 233) with a median fusion AVA of 0.92 cm^2^ (IQR 0.79–1.04 cm^2^) and fusion AVAi of 0.49 cm^2^/m^2^ (IQR 0.43–0.54 cm^2^/m^2^) (Tab. 2).

**Table 2:** Reclassification of aortic stenosis severity based on indexed fusion aortic valve area (AVAi) in the overall cohort (<0.60 cm^2^/m^2^: severe; AVAi ≥0.6 cm^2^/m^2^: moderate)

	Overall cohort	Patients reclassified to severe AS	Patients reclassified to moderate AS	*p*-value
	*n* = 359	*n* = 233	*n* = 126	
*Fusion aortic valve area (cm*^*2*^*), median (IQR)*	1.02 (0.87–1.21)	0.92 (0.79–1.04)	1.28 (1.16–1.46)	<0.001
*Indexed fusion aortic valve area (cm*^*2*^*/m*^*2*^*), median (IQR)*	0.54 (0.47–0.65)	0.49 (0.43–0.54)	0.70 (0.65–0.80)	<0.001
*Demographics*				
Age (years), median (IQR)	80 (75–84)	81 (76–84)	80 (75–84)	0.81
Male, *n* (%)	206 (57)	133 (57)	73 (58)	0.88
Height (cm), median (IQR)	168 (162–175)	170 (163–176)	167 (160–174)	0.060
Weight (kg), mean ± SD	76 ± 14	78 ± 13	72 ± 13	<0.001
Body mass index (kg/m^2^), median (IQR)	26 (24–29)	27 (24–30)	25 (23–28)	<0.001
Body surface area (m^2^), median (IQR)	1.9 (1.7–2.0)	1.9 (1.8–2.0)	1.8 (1.7–1.9)	<0.001
*Cardiac risk factors*				
Diabetes mellitus, *n* (%)	125 (35)	89 (38)	36 (29)	0.068
Hypertension, *n* (%)	271 (76)	171 (73)	100 (79)	0.21
*Medical history*				
Previous cerebrovascular accident or transient ischaemic attack, *n *(%)	75 (21)	52 (22)	23 (18)	0.37
Previous myocardial infarction, *n* (%)	107 (30)	57 (25)	50 (40)	0.003
Previous coronary artery bypass graft surgery, *n* (%)	110 (31)	64 (28)	46 (37)	0.081
Previous percutaneous coronary intervention, *n* (%)	157 (44)	100 (43)	57 (45)	0.67
Permanent pacemaker, *n* (%)	49 (14)	33 (14)	16 (13)	0.70
Peripheral vascular disease, *n* (%)	187 (52)	122 (52)	65 (52)	0.89
Chronic obstructive pulmonary disease, *n* (%)	104 (29)	64 (28)	40 (32)	0.37
Pulmonary hypertension, *n* (%)	60 (17)	34 (15)	26 (21)	0.14
Atrial fibrillation, *n* (%)	121 (34)	76 (33)	45 (36)	0.55
New York Heart Association class ≥III, *n* (%)	269 (75)	181 (78)	88 (70)	0.087
*Echocardiography*				
Left ventricular ejection fraction (%), mean ± SD	46 ± 15	46 ± 15	47 ± 16	0.39
Peak velocity (m/s), median (IQR)	3.5 (3.2–3.9)	3.7 (3.4–3.9)	3.3 (3.0–3.5)	<0.001
Peak gradient (mm Hg), median (IQR)	49 (41–61)	55 (46–61)	43 (35–51)	<0.001
Mean gradient (mm Hg), median (IQR)	29 (24–35)	32 (27–36)	25 (19–30)	<0.001
Aortic valve area (cm^2^), median (IQR)	0.8 (0.7–0.9)	0.7 (0.6–0.8)	0.9 (0.8–1.0)	<0.001
Indexed aortic valve area, median (IQR)	0.4 (0.4–0.5)	0.4 (0.3–0.4)	0.5 (0.5–0.6)	<0.001
LVOT VTI, median (IQR)	18 (15–21)	17 (16–21)	19 (16–22)	0.002
Aortic valve VTI, mean ± SD	79 ± 15	83 ± 14	72 ± 14	<0.001
Stroke volume index, median (IQR)	32 (26–40)	31 (26–38)	36 (28–43)	<0.001
Mitral regurgitation grade ≥II, *n* (%)	84 (23)	47 (20)	37 (29)	0.050
Aortic regurgitation grade ≥II, *n* (%)	51 (14)	30 (13)	21 (17)	0.33
*Multi-slice computed tomography*				
Annulus				
– Minimum diameter (mm), median (IQR)	22 (21–24)	22 (20–23)	23 (21–24)	<0.001
– Maximum diameter (mm), median (IQR)	27 (26–29)	27 (25–29)	28 (26–30)	0.003
– Mean diameter (mm), median (IQR)	25 (23–26)	25 (23–26)	25 (24–27)	0.001
– Area (mm^2^), median (IQR)	464 (413–525)	454 (402–516)	483 (437–546)	0.002
– Perimeter (mm), median (IQR)	78 (74–84)	77 (72–83)	80 (76–85)	0.001
– Aortic root calcification ≥ moderate, *n* (%)	259 (72)	166 (71)	93 (74)	0.61
– Calcium score (Agatston), median (IQR)	2121 (1320–3140)	2282 (1516–3345)	1833 (1184–2904)	0.002
Left ventricular outflow tract				
– Area (mm^2^), median (IQR)	448 (389–515)	432 (373–499)	467 (424–561)	<0.001
– LVOT calcification ≥ moderate, *n* (%)	46 (13)	34 (15)	12 (10)	0.17
*Risk score*				
Logistic EuroSCORE, median (IQR)	16 (10–24)	15 (10–22)	19 (11–27)	0.005

Values are expressed as median (interquartile range, IQR), *n* (%) or mean ± SD

*AS* aortic stenosis, *LVOT* left ventricular outflow tract, *VTI* velocity time integral

Fusion-based reclassification to moderate AS occurred in 35% of patients (*n* = 126) with a median fusion AVA of 1.28 cm^2^ (IQR 1.16–1.46 cm^2^) and median fusion AVAi of 0.70 cm^2^/m^2^ (IQR 0.65–0.80 cm^2^/m^2^) (Tab. 2). Reclassification of AS severity was also confirmed by using a calcium score which showed a median score of 1833 (1184-2904) in patients reclassified to moderate AS versus 2282 (1516-3345) in severe AS (*p* = 0.002, Tab. 2). Median Agatston score in women who were reclassified to moderate AS was 1371 (747-2289) versus 1572 (1047-2353)in women with severe AS (*p* = 0.18). Median Agatston score in men who were reclassified to moderate AS was 2382 (1519-3280) versus 2944 (2088-3935) in men with severe AS (*p* = 0.002). Patients reclassified to moderate AS had a lower BMI, more often a history of myocardial infarction, a higher logistic EuroSCORE and larger aortic annulus and LVOT dimensions on MSCT and TTE (Tab. 2). LVEF was <50% in 65 patients (52%) of patients reclassified to moderate AS.

### Clinical outcome

#### Overall cohort

Overall, 21 (6%) and 60 (17%) patients died within 30 days and 1 year after TAVI. Most deaths were due to a cardiac cause. Ten (3%) and 32 (9%) patients needed a rehospitalisation for HF within 30 days and 1 year after TAVI.

#### Reclassification of AS and outcome

**Fig. 1:**
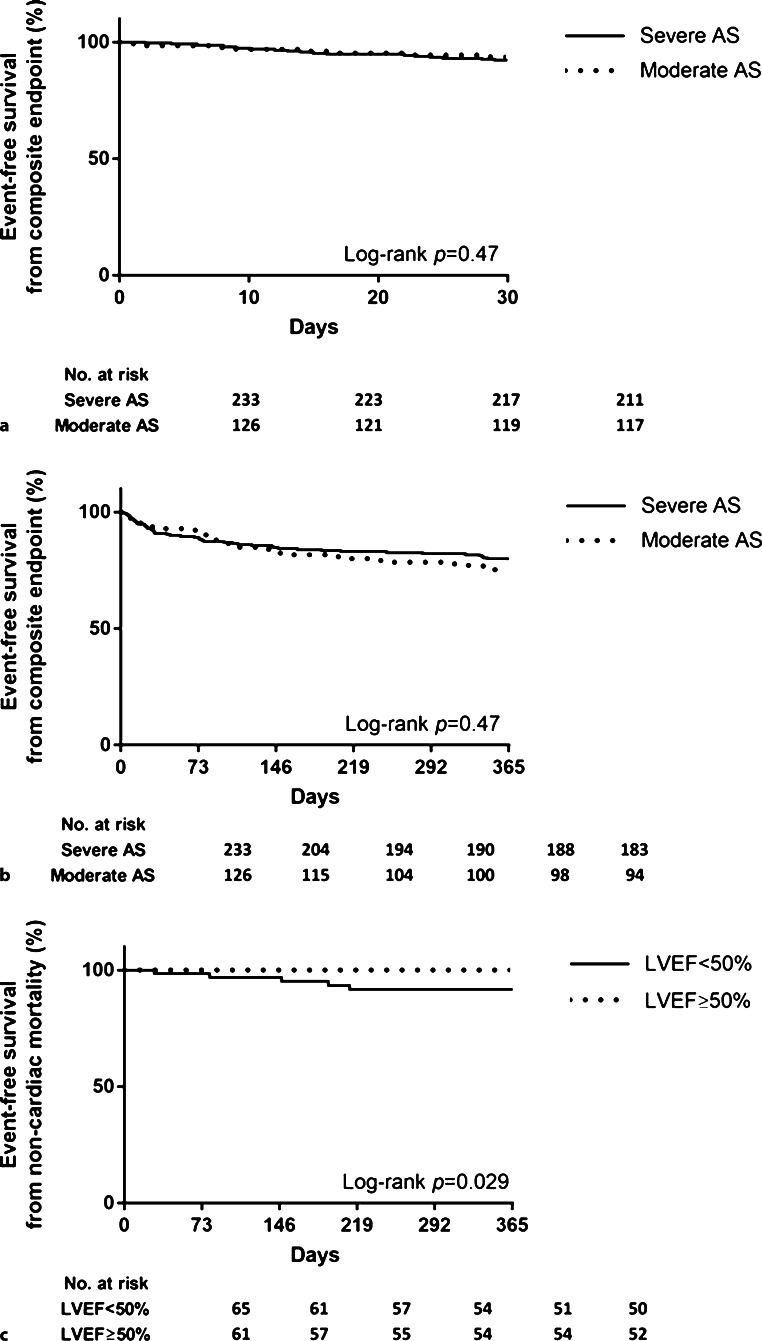
**a** Kaplan-Meier curve of primary endpoint (composite of all-cause mortality and heart failure rehospitalisations) in the overall cohort with comparison between patients reclassified to moderate and severe aortic stenosis 30 days after transcatheter aortic valve implantation. **b** Kaplan-Meier curve of primary endpoint (composite of all-cause mortality and heart failure rehospitalisations) in the overall cohort with comparison between patients reclassified to moderate and severe aortic stenosis at 1 year post-TAVI. **c** Kaplan-Meier curve of non-cardiac mortality 1 year post-TAVI in patients reclassified to moderate aortic stenosis with comparison based on baseline left ventricular ejection fraction (*LVEF*)

**Fig. 2:**
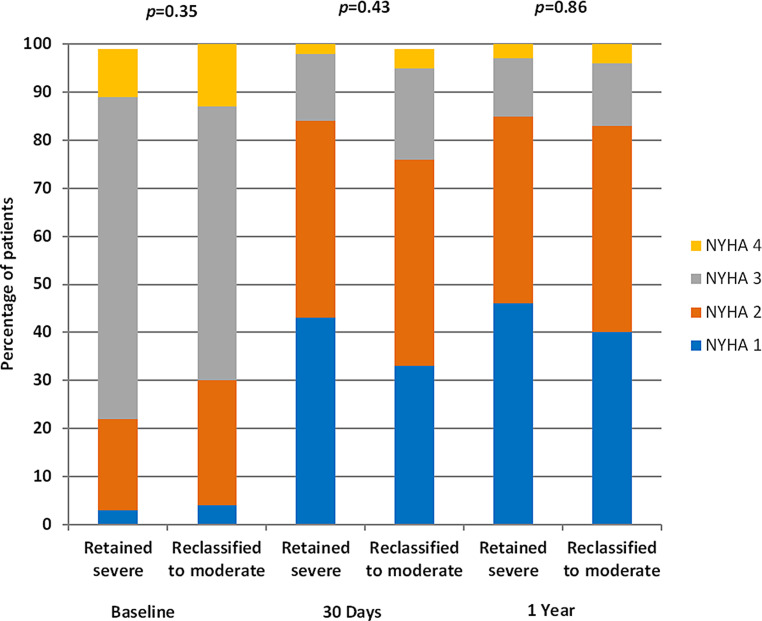
New York Heart Association (*NYHA*) class comparison between patients reclassified to moderate aortic stenosis or retaining severe aortic stenosis over time after transcatheter aortic valve implantation

The number of events for the primary endpoint (composite of all-cause mortality and HF rehospitalisations) was similar between patients reclassified to moderate AS and patients with severe AS at 30 days and 1 year post-TAVI (log-rank *p* = 0.47 and 0.47, Fig. 1a, b). There were no differences in cardiac or non-cardiac mortality or HF rehospitalisations during 1 year (log-rank *p* = 0.75 and 0.23, log-rank *p* = 0.35 and 0.67, log-rank *p* = 0.66 and 0.95, respectively). The LVEF remained stable at 1 year in patients reclassified to moderate AS and patients with severe AS (Tab. 3). Both cohorts showed similar improvements after TAVI in terms of New York Heart Association (NYHA) functional class (Fig. 2).

**Table 3:** Left ventricular ejection fraction over time (baseline vs 1 year post-TAVI)

	Baseline	1 year post-TAVI	Difference, mean (95% CI)	*p*-value
Left ventricular ejection fraction in the overall cohort (%), mean ± SD	46.40 ± 15.13	46.44 ± 12.94	0.040 (−1.67;1.75)	0.96
Left ventricular ejection fraction in severe AS (%), mean ± SD	45.42 ± 14.56	46.42 ± 12.59	1.00 (−1.14; 3.14)	0.36
Left ventricular ejection fraction in reclassified moderate AS (%), mean ± SD	48.21 ± 16.07	46.47 ± 13.66	1.74 (−4.62; 1.13)	0.23

*AS* aortic stenosis, *CI* confidence interval, *TAVI* transcatheter aortic valve implantation

#### Interaction between reclassified moderate AS and LVEF

Among patients reclassified to moderate AS, the number of events for the primary and secondary endpoints was similar in patients with LVEF <50% or ≥50%. Only non-cardiac death was more frequent in patients with LVEF <50% compared to LVEF ≥50% 1 year post-TAVI (log-rank *p* = 0.029, Fig. 1c).

## Discussion

The main results of the present study can be summarised as follows: (1) 35% of patients who underwent TAVI for severe AS with a low mean gradient (<40 mm Hg) were reclassified to moderate AS based on fusion AVAi; (2) the primary composite endpoint of all-cause mortality and HF rehospitalisations post-TAVI was similar in patients reclassified to moderate AS and patients with severe AS; (3) patients reclassified to moderate AS showed similar improvements in NYHA class compared to those with severe AS; (4) in patients reclassified to moderate AS non-cardiac death was more frequent in patients with LVEF <50% than in those with LVEF ≥50%.

In our study 35% of patients with low-gradient, severe AS were reclassified to moderate AS compared to 52% with normal-flow, low-gradient AS and 12% with low-flow, low-gradient AS in an earlier report [6]. Fusion AVAi results in the reclassification of a substantial number of patients with low-gradient, severe AS to moderate AS, particularly in the presence of normal flow [6]. Fusion imaging could be a valuable addition in the diagnostic work-up of patients with (low-gradient) AS, given the challenges in making an accurate diagnosis, although we could not correlate reclassification by this fusion technique to clinical outcome [14, 15]. In line with previous studies, our data confirmed the value of a CT-derived aortic valve calcium score to distinguish between moderate and severe AS (median of 1833 vs 2282, *p* = 0.002, Tab. 2; [16–18]).

In our study, patients reclassified to moderate AS had a lower BMI and BSA. This finding is surprising since it could be assumed that obese patients may have more challenging acoustic windows that may lead to inaccurate measurements of the LVOT [19]. On the other hand, it may be hypothesised that the practical challenges involved in obtaining satisfactory images in obese patients may require a more comprehensive study, performed by more experienced echocardiographers (operator selection bias). Nevertheless, a significantly lower BSA could further contribute to a larger AVAi.

Clavel et al. compared AVA based on LVOT measured by Doppler echocardiography (AVA_echo_) with AVA based on LVOT measured by CT (AVA_CT_) in 269 patients with AS [20]. Correlation between the aortic mean gradient and AVA was better with AVA_echo_ than with AVA_CT_ (*r* = −0.65 vs *r* = −0.61, respectively, *p* = 0.01). AVA_CT_ did not improve AS grading concordance or predict outcome. A similar survival outcome was observed under medical treatment with AVA_echo_ and AVA_CT_ (cut-off values 1.0 cm^2^ and 1.2 cm^2^ respectively) [20]. Our study corroborates these findings with no difference in clinical outcome in patients reclassified to moderate AS versus patients with severe AS. In addition, symptoms improved similarly in patients reclassified to moderate AS and in patients with severe AS. Whether a conservative (watchful waiting) approach in patients who were reclassified to moderate AS would have been equally safe cannot be inferred from our study. A propensity-matched analysis by Fougères et al. revealed similar long-term survival between patients with LV dysfunction and pseudo-severe (and thus moderate) AS treated conservatively versus HF patients with no AS [21].

Furthermore, our study demonstrated that TAVI in patients with LVEF <50% reclassified to moderate AS resulted in a similar clinical outcome at 1 year as in patients with severe AS and LVEF <50%. A recent multi-centre study of 305 patients with moderate AS and LV dysfunction (LVEF <50%) reported a 24% event rate of a composite of death, AVR or HF hospitalisation [22], which is comparable with the 29% rate of all-cause death and HF rehospitalisations in patients reclassified to moderate AS and LVEF <50% in this study. The randomised Transcatheter Aortic Valve Replacement to UNload the Left Ventricle in Patients With ADvanced Heart Failure (TAVR UNLOAD) trial (NCT02661451) is currently recruiting HF patients with moderate AS to evaluate the effect of TAVI on top of optimised HF treatment [23] and should shed further light on how to approach patients with this phenotype.

It is noteworthy that, in patients who were reclassified to moderate AS, LVEF <50% was associated with more non-cardiac deaths. Uncounted (non-cardiac) co-morbidities may partially explain the non-cardiac deaths. This observation may help to drive patient selection and suggests that, especially in patients with depressed LV function, the presence of non-cardiac co-morbidities may determine outcome rather than the moderate AS. Whether these patients would better not undergoing TAVI requires further study.

### Limitations

Our findings need to be interpreted in light of the retrospective study design and the modest sample size. Also, in the majority of cases, TAVI was performed with outdated transcatheter valve designs that may have affected early and later outcome. Indeed the introduction of sealing fabric and repositioning/retrievable features may improve transcatheter valve positioning, haemodynamic valve performance and mitigate paravalvular leakage. Intrinsic differences in echocardiography and CT imaging techniques precluded measurements at the exact same location in the LVOT. By consensus we made LVOT measurements by echocardiography 5 mm below the aortic annulus, whereas with CT we looked for the smallest area between 2 and 6 mm below the annular plane. This may have affected the reclassification results but seems inherent to the fusion concept. Also, we opted to measure the LVOT by echocardiography 5 mm below the annulus but recent insights may suggest that LVOT measurement precisely at the annular level may be more accurate because of the more circular configuration at this level [24]. The two centres analysed and provided their own data with no independent core laboratory assessment. The use of dobutamine stress echocardiography in our study was limited, and this may have resulted in misclassification of patients by TTE especially in the presence of LV dysfunction (pseudo-severe AS).

## Conclusion

The integration of CT and TTE to obtain the fusion AVAi resulted in the reclassification of approximately one third of patients with low-gradient AS to moderate AS. Reclassification did not affect clinical outcome, although patients reclassified to moderate AS with LVEF <50% had a worse outcome than patients with LVEF ≥50%.

## References

[1] Baumgartner H, Falk V, Bax JJ (2017). 2017 ESC/EACTS guidelines for the management of valvular heart disease. Eur Heart J.

[2] Nishimura RA, Otto CM, Bonow RO (2017). 2017 AHA/ACC focused update of the 2014 AHA/ACC guideline for the management of patients with valvular heart disease: a report of the American College of Cardiology/American HeartAssociation Task Force onClinical Practice Guidelines. J Am Coll Cardiol.

[3] Doddamani S, Grushko MJ, Makaryus AN (2009). Demonstration of left ventricular outflow tract eccentricity by 64-slice multi-detector CT. Int J Cardiovasc Imaging.

[4] Mehrotra P, Flynn AW, Jansen K (2015). Differential left ventricular outflow tract remodeling and dynamics in aortic stenosis. J Am Soc Echocardiogr.

[5] Ng AC, Delgado V, van der Kley F (2010). Comparison of aortic root dimensions and geometries before and after transcatheter aortic valve implantation by 2- and 3-dimensional transesophageal echocardiography and multislice computed tomography. Circ Cardiovasc Imaging.

[6] Kamperidis V, van Rosendael PJ, Katsanos S (2015). Low gradient severe aortic stenosis with preserved ejection fraction: reclassification of severity by fusion of Doppler and computed tomographic data. Eur Heart J.

[7] Kappetein AP, Head SJ, Généreux P (2012). Updated standardized endpoint definitions for transcatheter aortic valve implantation: the Valve Academic Research Consortium-2 consensus document. Eur Heart J.

[8] Baumgartner H, Hung J, Bermejo J (2009). Echocardiographic assessment of valve stenosis: EAE/ASE recommendations for clinical practice. Eur J Echocardiogr.

[9] Lang RM, Badano LP, Mor-Avi V (2015). Recommendations for cardiac chamber quantification by echocardiography in adults: an update from the American Society of Echocardiography and the European Association of Cardiovascular Imaging. Eur Heart J Cardiovasc Imaging.

[10] Schultz C, Moelker A, Tzikas A (2010). The use of MSCT for the evaluation of the aortic root before transcutaneous aortic valve implantation: the Rotterdam approach. EuroIntervention.

[11] Rosenhek R, Binder T, Porenta G (2000). Predictors of outcome in severe, asymptomatic aortic stenosis. N Engl J Med.

[12] Agatston AS, Janowitz WR, Hildner FJ, Zusmer NR, Viamonte M, Detrano R (1990). Quantification of coronary artery calcium using ultrafast computed tomography. J Am Coll Cardiol.

[13] Rodríguez-Olivares R, van Gils L, El Faquir N (2016). Importance of the left ventricular outflow tract in the need for pacemaker implantation after transcatheter aortic valve replacement. Int J Cardiol.

[14] Pibarot P, Messika-Zeitoun D, Ben-Yehuda O (2019). Moderate aortic stenosis and heart failure with reduced ejection fraction: Can imaging guide us to therapy?. JACC Cardiovasc Imaging.

[15] Clavel MA, Burwash IG, Mundigler G (2010). Validation of conventional and simplified methods to calculate projected valve area at normal flow rate in patients with low flow, low gradient aortic stenosis: the multicenter TOPAS (True or Pseudo Severe Aortic Stenosis) study. J Am Soc Echocardiogr.

[16] Cueff C, Serfaty JM, Cimadevilla C (2011). Measurement of aortic valve calcification using multislice computed tomography: correlation with haemodynamic severity of aortic stenosis and clinical implication for patients with low ejection fraction. Heart.

[17] Aksoy O, Cam A, Agarwal S (2014). Significance of aortic valve calcification in patients with low-gradient low-flow aortic stenosis. Clin Cardiol.

[18] Clavel MA, Pibarot P, Messika-Zeitoun D (2014). Impact of aortic valve calcification, as measured by MDCT, on survival in patients with aortic stenosis: results of an international registry study. J Am Coll Cardiol.

[19] Clavel MA, Burwash IG, Pibarot P (2017). Cardiac imaging for assessing low-gradient severe aortic stenosis. Jacc Cardiovasc Imaging.

[20] Clavel MA, Malouf J, Messika-Zeitoun D, Araoz PA, Michelena HI, Enriquez-Sarano M (2015). Aortic valve area calculation in aortic stenosis by CT and Doppler echocardiography. JACC Cardiovasc Imaging.

[21] Fougères E, Tribouilloy C, Monchi M (2012). Outcomes of pseudo-severe aortic stenosis under conservative treatment. Eur Heart J.

[22] van Gils L, Clavel MA, Vollema EM (2017). Prognostic implications of moderate aortic stenosis in patients with left ventricular systolic dysfunction. J Am Coll Cardiol.

[23] Spitzer E, Van Mieghem NM, Pibarot P (2016). Rationale and design of the Transcatheter Aortic Valve Replacement to UNload the Left ventricle in patients with ADvanced heart failure (TAVR UNLOAD) trial. Am Heart J.

[24] Hahn R, Pibarot P (2017). Accurate measurement of left ventricular outflow tract diameter: comment on the updated recommendations for the echocardiographic assessment of aortic valve stenosis. J Am Soc Echocardiogr.

